# Initial analysis of viral dynamics and circulating viral variants during the mRNA-1273 Phase 3 COVE trial

**DOI:** 10.1038/s41591-022-01679-5

**Published:** 2022-02-10

**Authors:** Rolando Pajon, Yamuna D. Paila, Bethany Girard, Groves Dixon, Katherine Kacena, Lindsey R. Baden, Hana M. El Sahly, Brandon Essink, Kathleen M. Mullane, Ian Frank, Douglas Denhan, Edward Kerwin, Xiaoping Zhao, Baoyu Ding, Weiping Deng, Joanne E. Tomassini, Honghong Zhou, Brett Leav, Florian Schödel, the COVE Trial Consortium

**Affiliations:** 1 Moderna, Inc., Cambridge, MA USA; 2 Brigham and Women’s Hospital, Boston, MA USA; 3 Baylor College of Medicine, Houston, TX USA; 4 Meridian Clinical Research, Omaha, NE USA; 5 University of Chicago, Chicago, IL USA; 6 University of Pennsylvania, Philadelphia, PA USA; 7 Clinical Trials of Texas, San Antonio, TX USA; 8 Criscor Clinical Research Institute, Medford, OR USA

**Keywords:** Drug development, Preventive medicine

## Abstract

The mRNA-1273 vaccine for coronavirus disease 2019 (COVID-19) demonstrated 93.2% efficacy in reduction of symptomatic severe acute respiratory syndrome coronavirus 2 (SARS-CoV-2) infections in the blinded portion of the Phase 3 Coronavirus Efficacy (COVE) trial. While mRNA-1273 demonstrated high efficacy in prevention of COVID-19, including severe disease, its effect on the viral dynamics of SARS-CoV-2 infections is not understood. Here, in exploratory analyses, we assessed the impact of mRNA-1273 vaccination in the ongoing COVE trial (number NCT04470427) on SARS-CoV-2 copy number and shedding, burden of disease and infection, and viral variants. Viral variants were sequenced in all COVID-19 and adjudicated COVID-19 cases (*n* = 832), from July 2020 in the blinded part A of the study to May 2021 of the open-label part B of the study, in which participants in the placebo arm started to receive the mRNA-1273 vaccine after US Food and Drug Administration emergency use authorization of mRNA-1273 in December 2020. mRNA-1273 vaccination significantly reduced SARS-CoV-2 viral copy number (95% confidence interval) by 100-fold on the day of diagnosis compared with placebo (4.1 (3.4–4.8) versus 6.2 (6.0–6.4) log_10_ copies per ml). Median times to undetectable viral copies were 4 days for mRNA-1273 and 7 days for placebo. Vaccination also substantially reduced the burden of disease and infection scores. Vaccine efficacies (95% confidence interval) against SARS-CoV-2 variants circulating in the United States during the trial assessed in this post hoc analysis were 82.4% (40.4–94.8%) for variants Epsilon and Gamma and 81.2% (36.1–94.5%) for Epsilon. The detection of other, non-SARS-CoV-2, respiratory viruses during the trial was similar between groups. While additional study is needed, these data show that in SARS-CoV-2-infected individuals, vaccination reduced both the viral copy number and duration of detectable viral RNA, which may be markers for the risk of virus transmission.

## Abstract (web summary)

The COVID-19 vaccine mRNA-1273 reduced SARS-CoV-2 copy number and duration of virus shedding, in addition to disease burden and infections, in exploratory analyses of breakthrough infections in the COVE trial.

## Main

The mRNA-1273 vaccine, a lipid nanoparticle-encapsulated messenger RNA vaccine encoding a prefusion-stabilized spike (S) protein of the prototype Wuhan-Hu-1 virus isolate, demonstrated high efficacy in prevention of symptomatic severe acute respiratory syndrome coronavirus 2 (SARS-CoV-2) infections in the primary analysis (December 2020) of the ongoing Coronavirus Efficacy (COVE) Phase 3 trial (clintrials.gov NCT04470427)^1^. Following emergency use authorization (EUA) issuance for mRNA-1273 in the United States, the trial was amended from the observer-blind part of the study (part A) to an open-label part B, and is currently ongoing until its completion after 2 years of follow-up. A recent update of the results reported after completion of the blinded portion of the study (March 2021), with 5.3 months of follow-up time, showed a vaccine efficacy (VE) rate of 93.2% against symptomatic infection and a safety profile similar to that observed in the primary efficacy analysis after 64 days^1,2^.

While SARS-CoV-2 vaccines are highly effective against symptomatic infection, and also reduce asymptomatic infections^2^, less is known about the effect of vaccination on the viral dynamics of disease, including the kinetics of viral copy number in the upper airways of persons with breakthrough infection, and the duration and magnitude of viral RNA shedding. Viral copy number quantitated by reverse transcription polymerase chain reaction (RT–PCR) in the upper airways is an important marker of SARS-CoV-2 infection, and its magnitude is related to the severity and symptoms of coronavirus disease 2019 (COVID-19), clinical outcomes and mortality, as well as SARS-CoV-2 transmission^3–8^. Viral copy number assessed by quantitative RT–PCR (RT–qPCR) may not accurately reflect SARS-CoV-2 infectious particles, in particular due to the detection of genome fragments and, although relationships between thresholds of high copy number and cultivable virus have been reported, additional studies are needed to more fully understand the usefulness of RT–PCR as a proxy for infectiousness and transmission^4,9–12^. Although genomic SARS-CoV-2 RNA is routinely detected by qualitative PCR and RT–qPCR in nasopharyngeal samples, detection of viral RNA copy number in saliva samples has generally comparable sensitivity and is a less-invasive, more convenient method for self-collection and serial sampling^13–15^. Recent prospective and retrospective studies have reported reductions in viral copy number and duration of viral shedding following COVID-19 vaccination as assessed by RT–PCR of nasopharyngeal samples^16,17^.

The emergence of several SARS-CoV-2 variants with mutations in the S protein genes and in other regions of the genome, and which exhibit decreased susceptibility to neutralization by infection and vaccine-induced antibodies, has raised the possibility of increased transmission and waning efficacy of current vaccines^18–27^. Variants include those previously considered to be variants of concern (VOC; Alpha (B.1.1.7), Beta (B.1.351) and Gamma (P.1)) and variants of interest (VOI; Eta (B.1.525), Iota (B.1.526), Kappa (B.1.617.1) and Epsilon (B.1.427, B.1.429)) that continue to be monitored, and current VOCs Delta (B.1.617.2 and AY lineages) and Omicron (B.1.1.529 and BA lineages)^28^. From initiation of the COVE trial (July 2020) to completion of the blinded portion of the study (data cutoff date 26 March 2021), the proportions of circulating VOCs and VOIs were very low in the United States and were predominantly of the Alpha, Beta, Epsilon and Iota lineages. Beginning in July 2021, the highly transmissible Delta (B1.617.2) VOC became the predominant cause of SARS-CoV-2 infections in the United States^28–30^. Higher viral copy number has been observed for Delta versus other variants in both vaccinated versus unvaccinated individuals, with similar to lower SARS-CoV-2 viral copy numbers and faster declines in viral copy number in those vaccinated^9–11,31^. Coinfection of SARS-CoV-2 with respiratory pathogens also occurs and can complicate the evaluation of SARS-CoV-2 infection rates, as well as patient management, due to the presence of nonspecific symptoms unrelated to COVID-19^32–34^.

The aim of this study was to assess the effect of mRNA-1273 vaccination on viral copy number and viral shedding, the burden of disease and infection and on viral variants detected in participants with COVID-19 by study group in the blinded part A of the COVE ongoing trial. We also explored the prevalence of SARS-CoV-2 viral variants and coinfecting respiratory pathogens up to May 2021 of the open-label part B of the study^1,2^.

## Results

### Effect of mRNA-1273 on SARS-CoV-2 genomic copy number

**Fig. 1:**
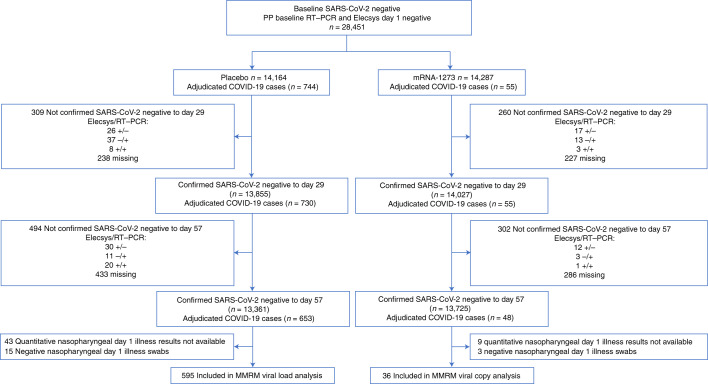
Viral copy and shedding analysis population. Included in the analysis were participants in the PP population who were SARS-CoV-2 negative by both binding antibody against NP (ROCHE Elecsys) and RT–PCR at baseline and day 29, and negative for binding antibody against NP (ROCHE Elecsys) at day 57 (ref. ^1^). The analysis was limited to adjudicated COVID-19 cases in the blinded portion of the study—that is, before unblinding or the data cutoff date of 26 March 2021, based on a database lock on 4 May 2021^1^.

SARS-CoV-2 genomic copy number was assessed in post hoc analyses of the cohort of participants with adjudicated COVID-19 cases that occurred at an illness visit (onset of participant COVID-19 symptoms and a positive virologic test by RT–PCR testing for SARS-CoV-2 by nasopharyngeal swab or, alternatively, if a clinic or home visit was not possible, by saliva (or nasal swab) samples, in the per-protocol (PP) population during the blinded and placebo-controlled phase of the COVE study^1,2^. The analysis population comprised adjudicated COVID-19 cases in the PP set with no evidence of infection from baseline study day 1 to day 57 (that is, those with negative binding antibody (bAb) against nucleocapsid protein (NP; ROCHE Elecsys) and RT–PCR at baseline and study day 29, and negative bAb against NP at day 57). There was a total of 799 adjudicated cases starting 14 days after dose 2, with 744 in the placebo and 55 in the mRNA-1273 group (Fig. 1). Of the 799 adjudicated cases, 701 (48 in the mRNA-1273 and 653 in the placebo group) had no evidence of infection to day 57 (–3/+7 days) and were included in the analysis of viral copy number. Baseline demographics and characteristics were generally balanced for placebo and mRNA-1273 groups (Supplementary Table 1). The mean age was 49 years (range 18–87 years) and 50% were female.

**Fig. 2:**
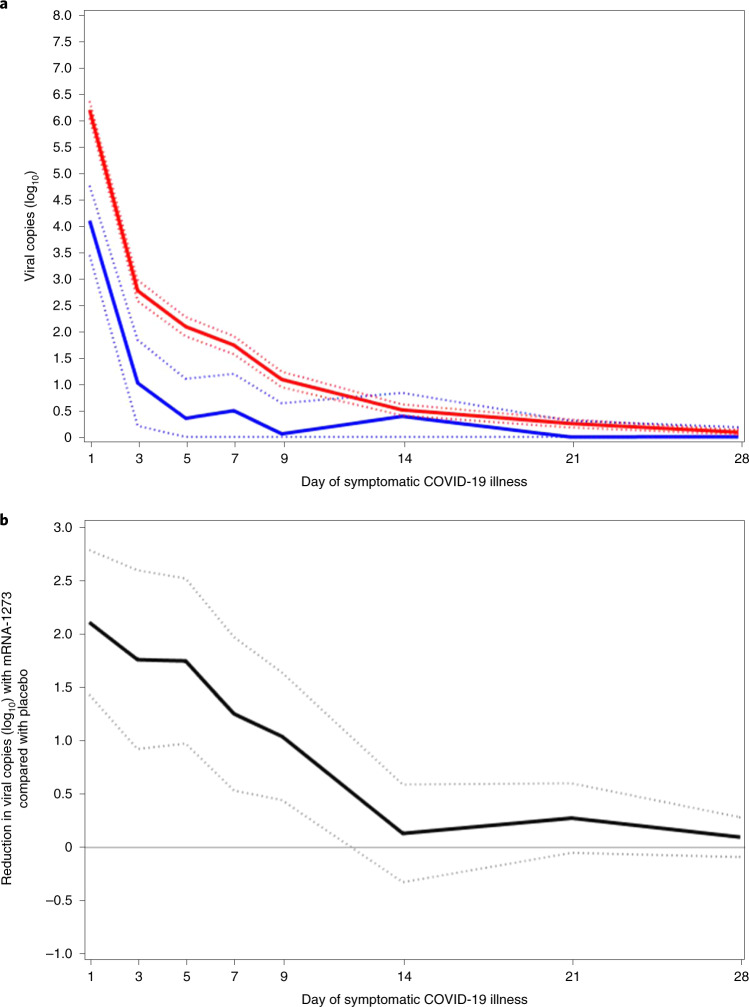
Reduction in SARS-CoV-2 viral copy number for mRNA-1273 compared with placebo. **a**,**b**, The number of viral copies (**a**) and reduction in number of viral copies (**b**) was assessed on the basis of SARS-CoV-2 RT–PCR Ct values converted to viral copy number (Methods). MMRM analysis was performed comparing absolute and change from baseline log_10_ viral copy between vaccinated and placebo participants, based on data from nasopharyngeal swabs at day 1 of illness and saliva samples at days 3, 5, 7, 9, 14, 21 and 28 of illness. Adjudicated cases in the blinded portion of the study were included. mRNA participants (*n* = 36) comprised 29 with first illness visits and 7 with second illness visits. Placebo participants (*n* = 595) included 527 cases from first illness visits and 61, 5 and 2 for second, third and fourth illness visits, respectively. **a**, Solid lines represent placebo (red) and mRNA-1273 (blue) and dotted lines correspondingly denote 95% CIs. **b**, Difference between mRNA-1273 and placebo participants in viral copies (log_10_, black solid line) and 95% CI (dotted lines).

**Extended Data Fig. 1:**
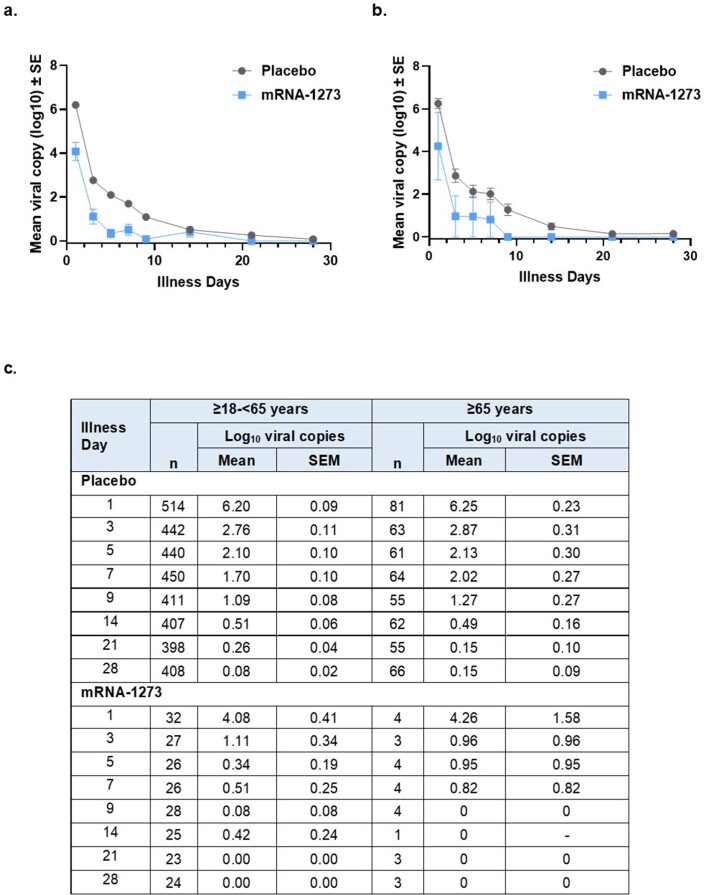
Viral copy number by age groups. Viral copy number (log_10_) in participants ≥18-<65 years (a) and ≥65 years (b) at the indicated illness day visits for placebo and mRNA-1273. Summary of log_10_ viral copies and mean and standard errors of the mean (SEM) for a and b plots (c).

**Extended Data Fig. 2:**
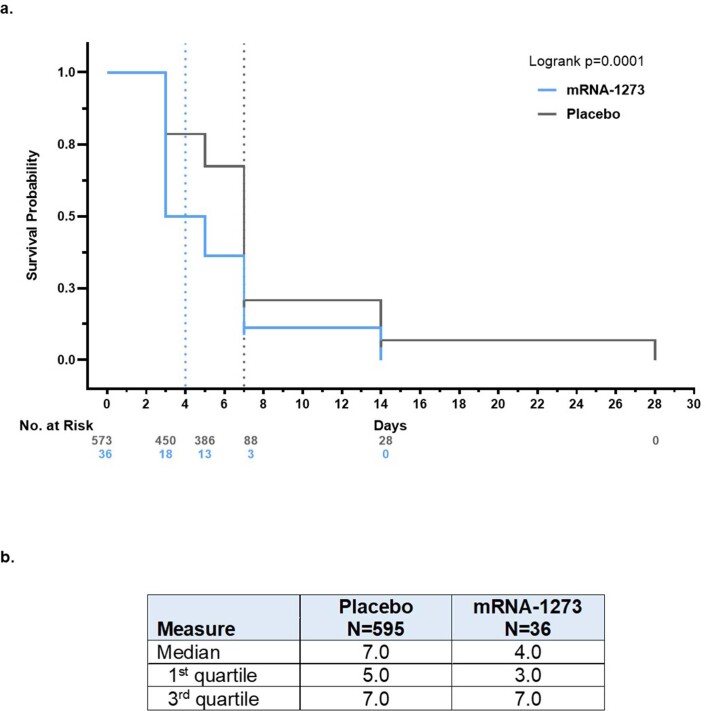
Time to undetectable SARS-CoV-2 viral copies. Survival probability of time to undetectable SARS-CoV-2 log_10_ viral copies for participants in placebo and mRNA-1273 groups (a). Dotted lines represent medians (days) for placebo (7) and mRNA-1273 (4). Tick marks indicate censored data. Nasopharyngeal swabs at day 1 of illness and saliva samples at days 3, 5, 7, 9, 14, 21, and 28 of illness. Lower limit of quantitation: <2.9 log_10_ copies/ml for saliva samples. Medians of time (days) to undetectable SARS-CoV-2 viral copies in (b).

Viral copy number was assessed by SARS-CoV-2 RT–qPCR and conversion of cycle-threshold (Ct) values to viral genome copy number. A mixed-model repeated-measures (MMRM) analysis was performed comparing absolute and change from baseline in log_10_ viral copy number between vaccinated and placebo participants from nasopharyngeal swabs at day 1 of illness and in saliva samples on days 3, 5, 7, 9, 14, 21 and 28 of illness. Only participants with a quantitative result available at the day 1 illness nasopharyngeal swab were included in the MMRM analysis. A total of 36 participants in the mRNA-1273 and 595 in the placebo group were included in the MMRM analysis of viral copy number. On illness visit day 1, the median number of viral copies per ml (log_10_) was 6.7 for the placebo group and 3.4 for the mRNA-1273 group (Supplementary Table 2). The MMRM analysis showed that, from days 1–9 of illness, the number of viral copies detected in the placebo arm was significantly higher (*P* < 0.001) than that in the mRNA-1273 arm (Fig. 2a and Supplementary Table 3). The difference (95% confidence interval (CI)) between the mRNA-1273 and placebo arms showed >100-fold reduction in viral copies per ml (log_10_) at day 1 (4.1 (3.4–4.8) versus 6.2 (6.0–6.4)), and a tenfold reduction at day 9 (0.06 (0–0.64) versus 1.1 (0.9–1.2)) in the mRNA-1273 group compared with the placebo group, respectively (Fig. 2b and Supplementary Table 4). Similarly, in age cohorts of participants 18 to <65 and ≥65 years of age, a 100-fold reduction in viral copy number at day 1 illness was observed for mRNA versus placebo (Extended Data Fig. 1). The median times to undetectable viral copies (lower limit of quantitation <2.85 log_10_ viral copies per ml) were 4 days for mRNA-1273 and 7 days for placebo (Extended Data Fig. 2).

### Burden of disease and burden of infection in the COVE trial

Exploratory analyses of burden of disease (BOD) and burden of infection (BOI) in COVE were performed in the blinded part A of the COVE trial, to assess the effect of vaccination on the severity and symptoms of COVID-19 (Supplementary Table 5). BOD was assessed in the PP set based on adjudicated cases in participants who had no evidence of SARS-CoV-2 infection, by serology and RT–PCR at randomization, and for whom postbaseline data were available. BOD was defined to reflect the severity of symptoms as a set of predefined scores (0, without COVID-19; 1, COVID-19 without hospitalization; 2, COVID-19 with hospitalization; and 3, death). mRNA-1273 vaccination substantially reduced COVID-19 illness with and without hospitalizations, and COVID-19 deaths. The mean (s.d.) of BOD scores was higher for the placebo (0.10 (0.24)) than the mRNA-1273 (<0.1 (0.07)) group, with a resulting VE point estimate (1 – ratio of mean BOD score) for mRNA-1273 on BOD of 93.2% (95% CI 91.0–94.8%). BOI was evaluated in the PP set based on asymptomatic infections and adjudicated COVID-19 symptomatic cases in participants who were SARS-CoV-2 infection negative at baseline and for whom postbaseline data were available. BOI score was defined similarly to that of BOD and additionally included asymptomatic infection (0, uninfected; 1/2, asymptomatic infection; 1, COVID-19 without hospitalization; 2, COVID-19 with hospitalization; and 3, death). Based on BOI score, vaccination reduced asymptomatic infections as well as COVID-19 with and without hospitalization. The mean (s.d.) of BOI scores was higher in placebo recipients (0.06 (0.24)) than in mRNA-1273 (0.01 (0.07)) recipients, with a VE vaccine of 91.2% (95% CI 89.0–93.0%). BOD and BOI scores were similar regardless of age and COVID-19 risk.

### SARS-CoV-2 variants detected in all COVID-19 cases in COVE

**Fig. 3:**
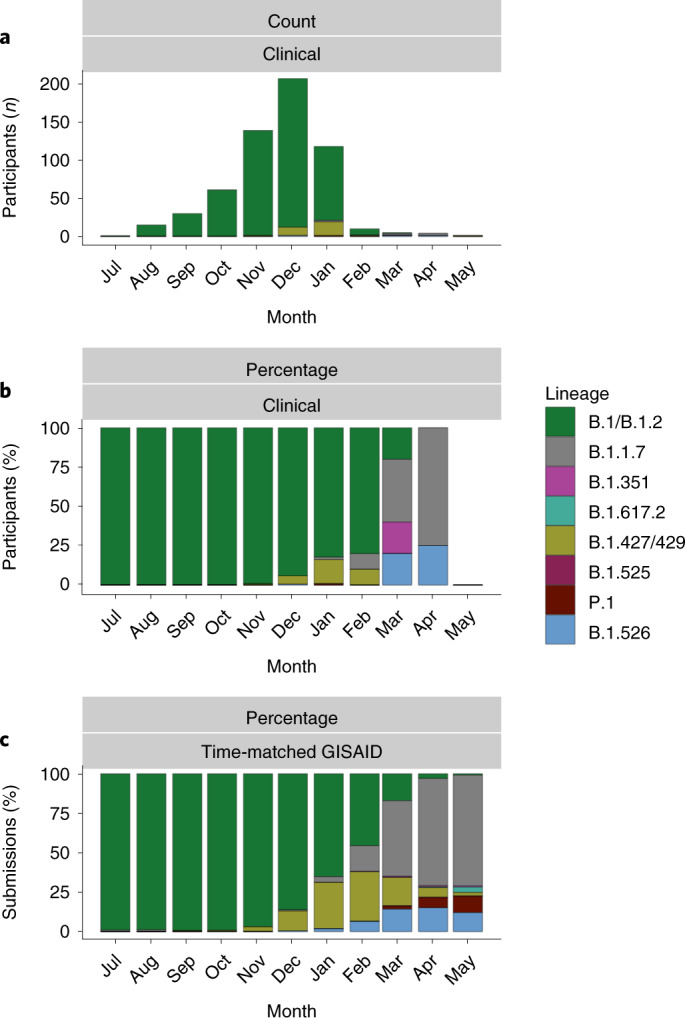
Spike-sequence-associated lineages found among COVE trial participants. **a**–**c**, Summary of selected spike-sequence-associated lineages found among COVE trial participants (regardless of symptoms) between July 2020 and May 2021. **a**, Number of sequences in the clinical dataset. **b**, Percentage of assigned lineages in the clinical dataset. **c**, Percentage of lineages circulating in the United States over the same period (time-matched sequence set) obtained from GISAID^30,36^.

Sequence information was obtained by RT–PCR from nasopharyngeal samples positive for SARS-CoV-2 collected in July 2020 from participants with COVID-19 in the blinded, placebo-controlled portion of the COVE trial, and extended into the open-label portion of the trial through May 2021 which was conducted in the United States^1^. This included all COVID-19 cases with positive tests, regardless of adjudication. There was a total of 1,006 samples available for sequencing (142 for mRNA-1273 and 864 for placebo). Although sequences could not be obtained from all samples, particularly in the mRNA-1273 group as sequencing success diminished with lower copy number, data for the spike gene were generated from 832 different samples (754 placebo and 78 mRNA-1273), corresponding to 791 trial participants (720 from placebo and 71 from mRNA-1273). To assess the relative prevalence of key variant lineages detected in the clinical dataset, Pango Lineages^35^ were inferred for each isolate based on amino acid mutations detected in the spike gene (Supplementary Table 6). Comparison of the prevalence of selected lineages in the clinical study (*N* = 589 assigned samples) with those from a US time-matched subset of the Global Initiative on Sharing All Influenza Data (GISAID) database^36^ revealed that the sequences detected in clinical case samples reflected the strains circulating in the United States during the trial, with similar frequencies between clinical samples and the GISAID subset (Fig. 3 and Supplementary Tables 7 and 8). In the study dataset, the majority of variants were of the B.1/B.1.2 lineage (548 (93%)) and mainly detected between July 2020 and February 2021. In addition, variants of the Epsilon (B.1.427/429) lineage (32 (5.4%)), first identified in California, were detected mainly during the months of December 2020 and January 2021, and two (1.0%)) Alpha (B1.1.7) variants between March and April 2021 (Supplementary Table 7). Overall, the frequencies of variants corresponded to the timeframes of EUA of COVID-19 vaccines and amendment of the COVE study (23 December 2020) with transition to the open-label portion (for example, January to May 2021), and were also similar to those in the GISAID database.

### SARS-CoV-2 variants in adjudicated COVID-19 cases in COVE

Viral variant sequences detected (545 in placebo and 28 in mRNA-1273 group) among the 825 adjudicated COVID-19 cases (769 in placebo and 56 in mRNA-1273 group) in the trial starting after randomization in the PP set included 1 (0.1%) wild-type lineage in the placebo group, with the majority in the B.1.2 (394 (51.2%) and 13 (23.2%)), B.1.243 (23 (3.0%) and 1 (1.8%)) and B.1.596 (13 (1.7%) and 0) lineages in the placebo and mRNA-1273 group, respectively (Supplementary Table 9). Overall, 18 (2.3%) of adjudicated cases starting after randomization were attributed to Epsilon, Gamma and Zeta variants in the placebo group and 3 (5.4%) in the mRNA-1273 group (Table 1 and Supplementary Table 9). Of these, 15 (2.0%) were Epsilon variants first detected in California in the placebo group and 3 (5.4%) in the mRNA-1273 group, including 9 (1.2%) B.1.429 and 6 (0.8%) B.1.427 variants in the placebo and 3 (5.4%) B.1.429 in the mRNA-1273 group.

**Table 1:** SARS-CoV-2 variants identified in the COVE trial PP set starting after randomization

Sequence variant	Placebo	mRNA-1273	Total
	*n* = 14,164	*n* = 14,287	*n* = 28,451
COVID-19 adjudicated cases, *n* (%)	769	56	799
Epsilon, Gamma and Zeta^a^	18 (2.3)	3 (5.4)	21 (2.6)
Epsilon and Gamma^b^	16 (2.1)	3 (5.4)	19 (2.4)
Zeta^c^	2 (0.3)	0	2 (0.3)
Epsilon variants detected in California	15 (2.0)	3 (5.4)	18 (2.3)
B.1.429	9 (1.2)	3 (5.4)	12 (1.5)
B.1.427	6 (0.8)	0	6 (0.8)

^a^Epsilon B.1.427 and B.1.429 (formerly considered VOCs at the time of sequencing) first detected in California, Gamma P.1 (originally VOI at the time of sequencing) and Zeta P.2.

^b^Includes Gamma P.1 (one (0.1)) in mRNA-1273.

^c^Zeta P.2. Variants categorized according to CDC (https://www.cdc.gov/coronavirus/2019-ncov/cases-updates/variant-surveillance/variant-info.html#Concern).

**Extended Data Fig. 3:**
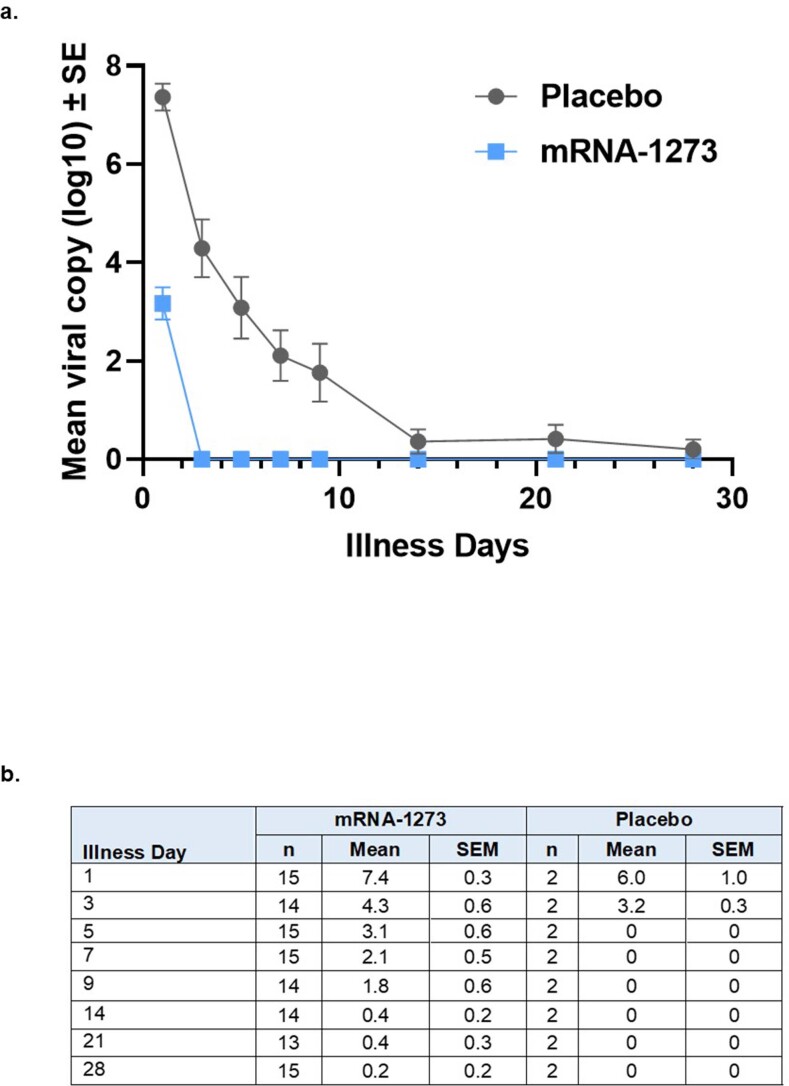
Viral copy number of variants first detected in California. Mean viral copy number (log_10_) of variants detected in participants over illness visit days (a) and summary of the means and standard errors of the mean (SEM) (b).

Exploratory analyses of blinded-phase data (cutoff date 26 March 2021)^2^ were performed to assess VE against specific variants in adjudicated COVID-19 cases starting 14 days after the second dose in the PP set. The competing risk method was used—specifically, Fine and Gray’s subdistribution hazard model based on COVID-19 cases with variants other than the specific VOI as competing risk. This exploratory analysis of variant-specific VE was performed for protection against Epsilon variants first detected in California (B.1.427 or B.1.429) because the total number of such cases was more than ten, and also against the formerly designated VOC and VOI given the interest in these variants^28,30^. For Epsilon variants first detected in California, VEs (95% CI) were 81.2% (36.1–94.5%) with 15 cases in the placebo and 3 in the mRNA-1273 group for combined variants B.1.427 and B.1.429 detected in California, and 68.9% (–12.8 to 91.4%; 9 in the placebo and 3 in the mRNA-1273 group) for the B.1.429 variant (Table 2). Based on the small number of former VOCs (Epsilon, Gamma and Zeta) detected in the placebo and mRNA-1273 groups (16 and 3, respectively), the VE (95% CI) to prevent COVID-19 by VOC was 82.4% (40.4–94.8%), and for VOI was 100.0% (not estimable—100.0%; 2 placebo and 0 mRNA-1273). Additionally, viral copies in cases with B.1.427 and B.1.429 variants detected were analyzed using MMRM. We observed >tenfold reduction in viral copies (copies per ml log_10_ (s.d.)) at the day 1 illness visit for mRNA-1273 (7.4 (0.3)) and placebo (6.0 (1.0)), and a shorter duration of detectable viral copies for mRNA-1273 versus placebo (Extended Data Fig. 3), consistent with the reduction in viral copy number associated with mRNA-1273 seen in overall cases regardless of the infecting variant.

**Table 2:** Exploratory analysis of vaccine efficacy against variants in the COVE trial PP set starting 14 days after the second dose

COVID-19 variant cases in COVE	Placebo	mRNA-1273
	*n* = 14,164	*n* = 14,287
COVID-19 primary efficacy endpoint^a^, *n* (%)	744 (5.3)	55 (0.4)
Vaccine efficacy based on hazard ratio (95% CI)^b^		93.2 (91.0–94.8)
Incidence rate per 1,000 person-years (95% CI)^c,d^	136.6 (127.0–146.8)	9.6 (7.2–12.5)
COVID-19 with Epsilon and Gamma variants, *n* (%)^a^	16 (0.1)	3 (<0.1)
Number with competing events, *n* (%)	728 (5.1)	52 (0.4)
Vaccine efficacy based on hazard ratio (95% CI)^b^		82.4 (40.4–94.8)
Incidence rate per 1,000 person-years (95% CI)^c,d^	2.9 (1.7–4.8)	0.52 (0.11–1.5)
COVID-19 with Zeta variant, *n* (%)^a^	2 (<0.1)	0
Number with competing events, *n* (%)	742 (5.2)	55 (0.4)
Vaccine efficacy based on hazard ratio (95% CI)^b^		100.0 (NE–100.0)
Incidence rate per 1,000 person-years (95% CI)^c,d^	0.38 (0.04–1.4)	–
COVID-19 with Epsilon variants first detected (CA), *n* (%)^a,e^	15 (0.1)	3 (<0.1)
Number with competing events, *n* (%)	729 (5.1)	52 (0.4)
Vaccine efficacy based on hazard ratio (95% CI)^b^		81.2 (36.1–94.5)
Incidence rate per 1,000 person-years (95% CI)^c,d^	2.8 (1.5–4.5)	0.52 (0.11–1.5)
COVID-19 with Epsilon (B.1.429) variant first detected (CA), *n* (%)^a^	9 (0.1)	3 (<0.1)
Number with competing events, *n* (%)	735 (5.2)	52 (0.4)
Vaccine efficacy based on hazard ratio (95% CI)^b^		68.9 (–12.8 to 91.4)
Incidence rate per 1,000 person-years (95% CI)^c,d^	1.7 (0.8–3.2)	0.52 (0.11–1.6)

Variants include Epsilon (B.1.427, B.1.429), Gamma (P.1) and Zeta (P.2). CA, California; NE, not estimable. COVID-19 cases with variant lineages other than the specified variant(s) assessed are considered as competing events.

^a^Based on participants with adjudicated assessments starting 14 days after second injection in the PP set, with censoring rules for efficacy analysis. If participant had a positive RT–PCR at the pre-dose 2 visit (day 29) without eligible symptoms for 14 days, or positive (Elecsys NP) at scheduled visits before becoming a COVID-19 case, the participant was censored at the date of positive RT–PCR or Elecsys NP.

^b^Vaccine efficacy, defined as 1 – hazard ratio (mRNA-1273 versus placebo), and 95% CI estimated using Fine and Gray’s subdistribution hazard model, with disease cases as competing events and treatment group as a covariate, adjusting for stratification factor.

^c^Person-years is defined as either total years from randomization date to the earliest among date of symptomatic SARS-CoV-2 infection, date of asymptomatic SARS-CoV-2 infection, last date of study participation and efficacy data cutoff.

^d^Incidence rate is defined as the number of participants with an event divided by the number of participants at risk and adjusted by person-years (total time at risk) in each treatment group; 95% CI was calculated using the exact method (Poisson distribution) and adjusted by person-years.

^e^Includes Epsilon variants B.1.427 and B.1.429, which were originally categorized as VOCs then reconsidered as VOIs and now as variants being monitored (VBM) by the CDC. Variant Gamma P.1 was formerly considered a VOC, but now a VBM.

### Respiratory pathogens detected in COVE at illness visits

**Fig. 4:**
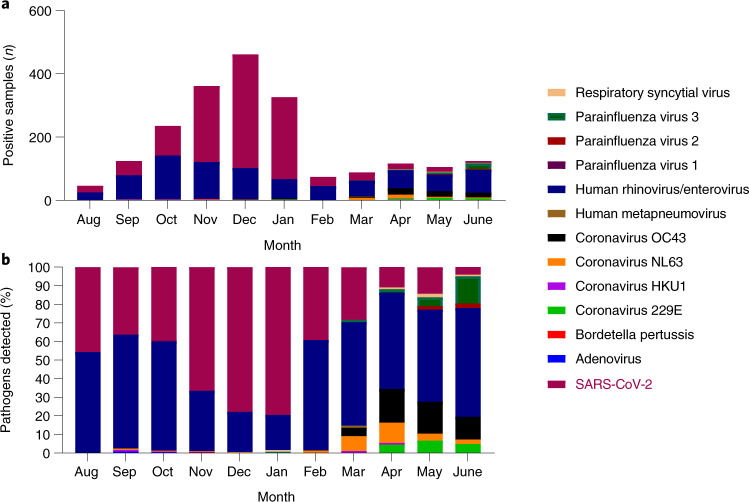
Respiratory pathogens detected in the COVE study, by month. **a**,**b**, Respiratory pathogen sequences detected from August 2020 to June 2021 in samples from all participant illness visits. **a**, Number of positive samples. **b**, Percentage of pathogens detected.

The presence of non-SARS-COV-2 viral and bacterial respiratory pathogens, in addition to SARS-COV-2, was detected between August 2020 and June 2021 in 954 total nasopharyngeal samples combined from placebo and mRNA-1273 recipients collected at illness visits, in parallel with SARS-CoV-2 testing in the placebo-controlled part A up to June 2021 of the open-label part B. From the initiation of the study in July 2020 to June 2021, 463 placebo participants had respiratory pathogens detected versus 491 mRNA-1273 participants. Participant illness visits peaked in December 2020, with 1,002 visits. That month, 461 participants tested positive for SARS-CoV-2 and/or other respiratory pathogens, with 78% attributed to SARS-CoV-2 and 22% to human rhinoviruses or enteroviruses. Coincident with EUA of COVID-19 vaccines by February 2021, the percentage of SARS-CoV-2 infections (regardless of symptoms) decreased to 39% of all positive results (*n* = 29/74) and, by June 2021, to 7% (*n* = 9/126; Fig. 4). As the rate of SARS-CoV-2 positivity decreased among participants along with the overall number of respiratory illness visits, the percentage of detection of other respiratory pathogens increased. Between August and December 2020, 62% (758) of all positive samples (*n* = 1,227) from illness visits were attributed to SARS-CoV-2 infections and 38% (469) to other respiratory pathogens, mainly rhinovirus/enterovirus infections, with 223 in the placebo versus 232 infections in the mRNA-1273 group. Between March and June 2021, 85% (365/431) of all positive illness results were attributed to respiratory pathogens other than SARS-CoV-2, the diversity of which increased through the spring months. There were 23 cases of coinfection with both SARS-CoV-2 and another respiratory pathogen between October 2020 and June 2021. The majority (19/23) were coinfections with human rhinoviruses or enteroviruses, and there were 3 cases of coinfection with a seasonal Coronavirus strain and 1 with human parainfluenza virus type 3 (HPIV3).

## Discussion

In this initial evaluation of the viral dynamics of SARS-CoV-2 infection in the ongoing COVE mRNA-1273 trial, vaccination with mRNA-1273, compared with placebo, significantly reduced the number of SARS-CoV-2 viral copies and duration of shedding in respiratory samples from participants with COVID-19. Consistent with the effect on viral copy number, vaccination with mRNA-1273 reduced the severity and symptoms of COVID-19 and SARS-CoV-2 infection in terms of hospitalizations and mortality, based on predefined BOD and BOI scores. Sequencing of the SARS-CoV-2 spike gene in clinical samples from the adjudicated cases in both the placebo and mRNA-1273 group in the trial revealed that variant lineages corresponded to the epidemiological patterns of circulating variants in the United States during the observation period, from the start of the blinded phase (July 2020) to June 2021 of the open-label portion. An exploratory analysis of SARS-CoV-2 VOC (Epsilon, Gamma and Zeta) and VOI (Zeta) detected in the placebo and mRNA-1273 groups resulted in estimated vaccine efficacies (95% CI) of 82.4% (40.4–94.8%) for VOCs, 100% for VOIs and 81.2% (36.1–94.5%) for variants Epsilon B.1.427 and B.1.429. In addition, the majority of symptomatic respiratory cases during the blinded, randomized portion of the trial were attributed to SARS-CoV-2 while the percentage of non-SARS-CoV-2 respiratory pathogens increased, coincidently with the availability of COVID-19 vaccines under EUA and crossover of placebo recipients to mRNA-1273 in the COVE study starting in January 2021.

Studies have shown that higher viral copy number, evaluated by RT–PCR and quantitated by Ct values (lower Ct values) and/or converted to copies per ml (as assessed in this study), is related to severe COVID-19 and mortality^3–8^, and at thresholds shown to be associated with cell culture infectivity^12,37^. COVID-19 vaccination has been shown to attenuate viral copy number and the duration of viral shedding and illness when assessed by RT–PCR^16,17^. In our study, the findings that mRNA-1273 elicited a highly significant reduction in both viral copy number on the day of COVID-19 illness diagnosis and persistence of viral RNA in saliva samples indicative of viral shedding up to day 9 are relevant as potential surrogates for the transmissibility of the SARS-CoV-2 virus^3,5,12^. In line with these findings and the efficacy results reported at the end of the blinded part of the COVE trial^1,2^, mRNA-1273 reduced the symptoms and severity of disease and infection as reflected by the lower BOD and BOI scores seen in vaccine recipients, and this is consistent with recent studies showing that vaccination is highly effective in prevention of severe COVID-19, COVID-19-related hospitalizations and deaths, and also reduces asymptomatic infection^38–41^. Although the transmission dynamics of COVID-19 are still under study, the estimated ten- to 100-fold reductions in viral copy number, with statistically significant reductions in copies to day 9, may enable a substantial reduction in COVID-19 disease spread, even in vaccine breakthrough cases.

The high efficacy of mRNA-1273 was originally demonstrated during a period of relatively lower diversity of circulating strains (July to December 2020) that corresponded to the bulk of the blinded and placebo-controlled phase of the trial to March 2021, and the number of breakthrough cases remained low through June 2021 of the open-label phase until the surge of Delta variants in July 2021^42^. The efficacy data from this exploratory analysis of specific variants of COVID-19 cases, combined with the available respiratory pathogen data to June 2021, suggest that high VE was maintained during the timeframe, which encompassed not only the peak of the US epidemic (January 2021) but also the emergence of variants Alpha, Beta, Epsilon and Gamma. Supported by the sequence data, an exploratory analysis of the VE of mRNA-1273 against circulating variants was performed. Among all VOCs, a sufficient number of cases was accrued during the blinded phase to allow for a formal analysis only of the Epsilon variants first detected in California (B.1.427 and B.1.429), with a VE (95% CI) of 81.2% (36.1–94.5%) against variants B.1.427 and B.1.429. Thus, mRNA-1273 maintained high efficacy against the relatively small number of VOIs and VOCs circulating to April 2021, and appears slightly reduced compared with that of the wild-type lineage, albeit the numbers are insufficient for formal comparison^18–27^. While mRNA-1273 has remained a highly efficacious vaccine, there is a potential for variants to generate vaccine breakthroughs as more transmissible and divergent variants emerge, including Delta, amid potentially waning immune responses months after vaccination^24,28–30,42,43^.

Infection with SARS-CoV-2 causes a broad range of symptoms, from asymptomatic or mild symptoms to severe illness and death; however, coinfections with respiratory pathogens can also contribute to these symptoms, complicating diagnosis and patient management^32–34^. The finding that the majority of symptomatic respiratory cases were caused by SARS-CoV-2 infection, and mostly among placebo recipients during the blinded portion of the study, further confirms the high efficacy of mRNA-1273 vaccine in the prevention of symptomatic, molecularly confirmed COVID-19 disease^1,2^. The percentage drop in SARS-CoV-2 infection and increase in the percentage of non-SARS-CoV-2 respiratory pathogens among trial participants from February to June 2021, when participants in the placebo group began crossing over to receive mRNA-1273, is consistent with the high efficacy of the vaccine against SARS-CoV-2 at a time when relaxation of infection prevention measures resulted in the emergence of other respiratory viruses. The presence of other respiratory viruses was similar between the mRNA-1273 and placebo groups, indicating that case ascertainment between the two groups was unbiased, and also was not impacted by mRNA-1273 vaccination. The observations of high efficacy, combined with a reduced viral copy number in breakthrough cases and reduced burden of disease and infections, demonstrate that immunization with mRNA-1273 does not lead to vaccine-enhanced respiratory disease in the short term, a theoretical concern at the onset of COVID-19 vaccine development^1,2^.

The strengths of this report on SARS-CoV-2 viral dynamics are that the analyses were performed in a large blinded, randomized-controlled study of mRNA-1273 vaccine that extended through to June 2021 of the open-label part B for variant sequence and respiratory pathogen detection; however, there are several limitations to consider. First, it should be noted that the analyses presented here are based on an initial evaluation of data that will continue to accrue over time and will be subject to future updates. Although the BOD and BOI results were statistically significant over the entire PP set, the two scales have some redundancies. While the results were driven by the overall high vaccine efficacy and a numerically dominant prevalence of mild-to-moderate disease, including asymptomatic infections for BOI, these two analyses also reveal differences in an important measure of COVID-19 impact, hospitalization. The impacts of therapies used to manage cases of COVID-19 on the burden of disease, severity of disease and death were not assessed given that the study was blinded; however, it is unlikely that there were systematic differences in the use of standard of care between treatment groups over the course of the trial. With increased follow-up times and inevitably acquired disease in participants, as well as the introduction of new therapeutic interventions, the proportions of BOD and BOI may change; however, a prespecified, proportional means model was used for this analysis, to allow direct comparison across different periods of follow-up, minimizing these effects. The sequence data also have known caveats, some demonstrated through this work. For example, the fact that the vaccine has a marked effect on lowering SARS-CoV-2 viral copy number hampered our efforts to generate an unbiased sequence dataset. Even though sequencing efforts were performed in a blinded manner and the team was unaware of participant treatment assignments, the success rates in obtaining good-quality sequences from samples of vaccine breakthrough cases were 50% or less, but >80% for placebo-originated samples. Although this is a clear, unavoidable bias in the available sequence data, it does not affect the overall assessment of the biological effect of vaccination on viral copy number. We also did not test for the presence of infectious virus, although a relationship between high viral copy number and infectious virus has previously been demonstrated^12,37^. Although this analysis was conducted before the predominant circulation of Delta variants (July 2021) in the United States and an assessment of the full impact of mRNA-1273 efficacy on Delta requires additional studies, the vaccine has shown effectiveness against this variant^42^. We must also recognize that the small sample size of variants detected in this analysis limits the assessment and interpretation of VE against emerging variants. While highly aggressive variants capable of causing both higher numbers of cases and increased viral copy number among vaccine recipients were not seen during the timeframe of the analysis, the emergence of the Delta variant may yield samples that can be more readily sequenced in future analyses and, hence, a resulting detection bias in its favor^28,44^. A further limitation is that the treatment groups in the original 1:1 randomized blinded and placebo-controlled trial had evolved, as an increasing number of placebo recipients either crossed over to be immunized or left the study to seek vaccination outside of the study in the analyses of the prevalence of viral variants and respiratory pathogens.

In summary, this analysis of the viral dynamics and circulating viral variants in the mRNA-1273 Phase 3 COVE trial during the placebo-controlled phase suggests that vaccination with mRNA-1273 vaccine leads to a significant reduction in SARS-CoV-2 viral copy number and shedding period, and associated BOD and BOI. The mRNA-1273 vaccine showed high efficacy and continues to have a marked effect in reducing symptomatic COVID-19 cases among vaccine recipients. The shifting landscape in terms of new emerging variants, and the potential waning of the immune response, suggest that a continuation of these studies is warranted.

## Methods

### Trial design

This is an analysis of the previously reported COVE study, a Phase 3 randomized, observer-blinded, placebo-controlled trial that enrolled adults in medically stable condition at 99 US sites (clintrials.gov NCT04470427)^1,2^. Eligible participants included adults 18 years or older with no known history of SARS-CoV-2 infection and whose circumstances put them at appreciable risk for SARS-CoV-2 infection and/or high risk of severe COVID-19. Participants were randomized 1:1 to receive mRNA-1273 vaccine (100 µg) or placebo and were stratified by age and COVID-19 complications risk criteria (≥18 and <65 years of age and not at risk, ≥18 and <65 years and at risk, and ≥65 years). Following issuance of the EUA of mRNA-1273, the protocol was amended as a two-part Phase 3 study (parts a and b; Supplementary protocol). Part A was the observer-blinded-to-treatment phase, which concluded when participants unblinded and consideration was given those on placebo to receive mRNA-1273. Part B is the currently ongoing open-label phase. The blinded part of the trial was completed^2^. Participants will continue to be followed up for up to 2 years as originally planned.

The COVE trial is conducted in accordance with the International Council for Technical Requirements for Registration of Pharmaceuticals for Human Use, Good Clinical Practice Guidance and applicable government regulations. The central Institutional Review Board/Ethics Committee (Advarra) approved the protocol and consent forms. All participants provided written informed consent.

The design, efficacy assessments and study treatment of the COVE trial have previously been described and are provided in the Supplementary protocol online^1,2^. Briefly, the primary endpoint of the study was the vaccine efficacy of mRNA-1273 at preventing a first occurrence of symptomatic COVID-19 with onset ≥14 days post second injection, COVID-19 cases being defined as those having two or more systemic symptoms (fever ≥38 °C, chills, myalgia, headache, sore throat, new olfactory and taste disorder(s)) or experienced one or more respiratory signs or symptoms (cough, shortness of breath or clinical or radiological evidence of pneumonia) and confirmed by positive RT–PCR for SARS-CoV-2 using a NP swab, nasal or saliva sample. The secondary endpoint, severe COVID-19, was defined as confirmed COVID-19 as per the primary endpoint case definition, plus one of the clinical signs indicative of severe systemic illness (respiratory rate ≥30 min^–1^, heart rate ≥125 beats min^–1^, oxygen saturation (SpO_2_) ≤93% on room air at sea level or arterial oxygen partial pressure/fractional inspired oxygen (PaO_2_/FIO_2_) <300 mmHg, or respiratory failure or acute respiratory distress syndrome (defined as needing high-flow oxygen, noninvasive or mechanical ventilation or extracorporeal membrane oxygenation), evidence of shock (systolic blood pressure (BP) <90 mmHg, diastolic BP <60 mmHg or requiring vasopressors), or significant acute renal, hepatic or neurologic dysfunction, or admission to an intensive care unit or death). Safety assessments included evaluation of solicited local and systemic adverse events with onset during the 7 days following each injection to resolution, unsolicited adverse events during 28 days following each injection, adverse events leading to discontinuation from dosing and/or study participation, medically attended and serious adverse events throughout the study and severity graded as described in the protocol.

In this analysis of the COVE trial, viral variants were sequenced in all COVID-19 and adjudicated COVID-19 cases, viral copy number and shedding and vaccine efficacy against variants were assessed in adjudicated COVID-19 cases in the PP set from the blinded portion of the study (data cutoff 26 March 2021). Following amendment (23 December 2020) of the COVE Phase 3 study, the open-label phase of the trial was initiated and participants in the placebo arm started to receive the mRNA-1273 vaccine. Sequence data for viral variants, and also assessment of the presence of other respiratory pathogens, extended into the timeframe of the open-label part of the study.

### Study procedures

#### Assessment of viral copy number

##### Study design and population

The study population for analysis of viral copy number were those participants in the PP population who had received two doses of placebo or mMRA-1273 and had no evidence of infection from baseline at day 1 to day 57 of the study, defined as having negative baseline values (baseline SARS-CoV-2 negative) by binding antibody assay to NP (ROCHE Elecsy), and also had follow-up Elecsys-NP-negative values at days 29 and 57 of the study, and RT–PCR-negative results at baseline days 1 and 29. These specifications were applied to the analysis population to ensure that participants received both doses of the vaccine (or placebo) and were at risk for infection for measurement of viral load, providing a more homogeneous population of fully vaccinated individuals for calculation of viral load. The analysis period was limited to the blinded portion of the study, and the data cutoff date was 26 March 2021 (or earlier unblinding).

The number of SARS-CoV-2 RNA copies was assessed in the cohort of participants with adjudicated COVID-19 cases at an illness visit (onset of symptoms and virological test) in the PP population during the blinded and placebo-controlled phase of the COVE study^1,2^. During the study, an illness visit was scheduled if participants experienced the presence of prespecified symptoms including fever (temperature ≥38 °C) or chills (of any duration, including ≤48 h), shortness of breath or difficulty breathing (of any duration, including ≤48 h), cough (of any duration, including ≤48 h), fatigue, muscle or body aches, headache, new loss of taste or smell, sore throat, congestion or runny nose, nausea or vomiting, or diarrhea as per Centers for Disease Control and Prevention (CDC) guidelines lasting at least 48 h (except for fever and/or respiratory symptoms), at which a nasopharyngeal swab was collected within 72 h for SARS-CoV-2 RT–PCR testing. Alternatively, if a clinic or home visit was not possible, a saliva (or nasal swab) sample was submitted for SARS-CoV-2 RT–PCR testing. Study participants self-collected saliva samples at 3, 5, 7, 9, 14 and 21 days after the initial illness visit, and a saliva sample was also collected at a convalescent visit scheduled approximately 28 days after the initial illness visit.

In the COVE trial there was a total of 799 adjudicated cases starting 14 days after dose 2, with 744 in the placebo and 55 in the mRNA-1273 group. Of the 799 adjudicated cases, 701 (48 in the mRNA-1273 and 653 in the placebo group) had no evidence of infection to day 57 and were included in the analysis of viral copy number. Viral copy number was assessed by SARS-CoV-2 RT–qPCR, and Ct values were converted to viral genome copy number. A MMRM analysis was performed comparing absolute and change from baseline in log_10_ viral copy number between vaccinated and placebo participants from nasopharyngeal swabs at day 1 of illness, and in saliva samples on days 3, 5, 7, 9, 14, 21 and 28 of illness. Only participants with matching quantitative results available for the day 1 illness nasopharyngeal swab and saliva samples at days 3, 5, 7, 9, 14, 21 and 28 of illness were assessed in the MMRM analysis. The analysis included a total of 36 participants in the mRNA-1273 and 595 in the placebo group.

##### Statistical analysis

For the cohort of COVID-19 adjudicated cases, the day 1 illness nasopharyngeal swab and the days 3, 5, 6, 9, 14, 21 and 28 of illness saliva specimens were matched to assess the qualitative and quantitative results for each. SARS-CoV-2 RT–PCR was performed as described below (Eurofins Viracor). Conversion from Ct time to viral copies for the RT–qPCR was log_10_ viral copies per ml = Ct – 40.9578/–3.3385 for swabs (day 1) and log_10_ viral copies per ml = Ct – 41.0349/–3.3346) for saliva (days 3, 5, 7, 9, 14, 21 and 28). If the qualitative result was negative, log_10_ viral copies were assumed to be 0. MMRM analysis was performed comparing absolute and change from baseline log_10_ viral copy number between vaccinated and placebo participants from the nasopharyngeal swab on day 1 of illness through saliva sample days 3, 5, 7, 9, 14, 21 and 28 of illness. Only participants with a quantitative result for the day 1 illness nasopharyngeal swab were included in the MMRM modeling. Any MMRM result estimate <0 copies was truncated at 0. There was no imputation for missing data. SAS v.9.4 was used in the analysis. Proc mixed with the restricted maximum likelihood (reml) method, modeling log_10_ viral copy with the covariates of treatment, illness day and the treatment by illness day interaction term was performed. Illness day was the repeated measure by participants, and the variance structure was unrestricted. Least-squares means differentials were used to generate the contrast statements.

#### Vaccine efficacy against BOD

An exploratory analysis of BOD due to COVID-19 was performed in the PP population of the COVE trial based on adjudicated cases in participants who were SARS-CoV-2-naîve by serology and PCR at randomization, and had available postbaseline data. A BOD score was based on post-SARS-CoV-2 infection follow-up and defined to reflect the severity of symptoms (0, without COVID-19; 1, COVID-19 without hospitalization; 2, COVID-19 with hospitalization; 3, death). A summary of BOD score and the number and percentage of participants with each level of BOD score are provided for the treatment group. To assess disease burden in participants with COVID-19, a summary of BOD was provided for participants with COVID-19 (that is, those with BOD = 0 were excluded from the analysis) and, for assessment of the impact of baseline risk of severe disease on vaccine effect regarding disease severity, a summary of BOD was provided by randomization strata (≥65 years, <65 years and at risk and <65 years and not at risk). A proportional means model, including treatment group as fixed effect and stratified with the stratification factor at randomization, was used to assess the vaccine effect on BOD. The VE for BOD scores was estimated as 1 – the ratio of means as estimated by the proportional means model based on weighted scores and reported with 95% CIs.

#### Vaccine efficacy against BOI

An exploratory analysis of BOI was performed in the PP set based on asymptomatic infections and adjudicated COVID-19 symptomatic cases in participants who were SARS-CoV-2 infection negative at baseline and had available postbaseline data. As with BOD, a BOI score was defined to assess the severity of symptoms (0, no infection; 1/2, asymptomatic infection; 1, COVID-19 without hospitalization; 2, COVID-19 with hospitalization; 3, death). A proportional means model, including treatment group as fixed effect and stratified with the stratification factor at randomization, was used to assess the vaccine effect on BOI. The VE for BOI was estimated as 1 – the ratio of mean BOI scores and reported with 95% CIs.

#### Sequencing methodology and sequence data analyses

Sequencing of the SARS-CoV-2 spike gene was attempted from all available SARS-CoV-2 RT–PCR-positive nasopharyngeal samples (*n* = 832) collected between July 2020 and May 2021 from participants in the blinded portion of the COVE trial. This corresponded to 791 participants (*n* = 720 placebo and *n* = 71 mRNA-1273). For 41 participants, more than one sample was sequenced within the illness period. Analysis of these cases is ongoing and will be included in additional analyses/reporting at a later date. Sequencing data were generated using three different approaches in two different laboratories.

##### Spike gene sequencing assay from Eurofins Viracor

Viral RNA from nasal swabs was extracted using the NucliSENS easyMag extraction kit. Extracted RNA was used as template in a Qiagen One-Step Reverse Transcription-PCR (RT–PCR) reaction for complementary DNA synthesis using SARS-CoV-2 S gene Conventional RT–PCR primer mixes. Either the Agilent 2200 or TapeStation 4200 controller, in conjunction with D5000 ScreenTapes, D5000 reagents and the TapeStation Analysis software A.02.02, was used to assess postamplified and purified PCR reactions for the presence, size and concentration of any products generated. Library preparation was performed using the Illumina Nextera XT Library Prep Kit. Either the Agilent 2200 or 4200 TapeStation, in conjunction with D5000 ScreenTapes, D5000 reagents and the TapeStation Analysis software, was used to assess purified libraries for the presence, average fragment size and concentration of fragment distributions generated. Analysis of next-generation sequencing (NGS) data for the SARS-CoV-2 S gene NGS assay was done using Qiagen CLC Genomics Workbench v.20.0.1, with NC_045512.2 as the reference strain. Custom workflow in the CLC Genomics Workbench processed the sequencing data as follows: paired fastq files were imported, primer sequences were trimmed from the 5'-ends of reads, reads were mapped to the full SARS-CoV-2 reference genome (NC_045512.2), single-nucleotide and insertion/deletion variants relative to reference were called and annotated and a consensus sequence of the spike gene (bases 21615–25436) was generated. The analysis workflow reported annotated variant tables, spike gene coverage tables and spike gene consensus sequences.

Following TapeStation D5000 assessment and subsequent analysis of data using TapeStation Analysis software A.02.02, if the viral copy number was insufficient to obtain a correct band for SARS-CoV-2 S gene targets (S1 (1,026 base pairs (bp)), S2 (893 bp), S3 (1,178 bp) and S4 (1,264 bp)), these results were considered negative. Positive results for RT–PCR reactions were identified by (1) the presence of a band of the appropriate size for SARS-CoV-2 S gene PCR products (S1 (1,026 bp), S2 (893 bp), S3 (1,178 bp) and S4 (1,264 bp) relative to the D5000 ladder); and (2) a peak table reporting a concentration for bands specific to the sample. SARS-CoV-2 S gene NGS runs using MiSeq v.2 chemistry reagents running paired-end 2 × 151 reads must exhibit the following criteria: cluster densities approximating 600–1,200 K mm^–2^ and >80% of bases called exhibiting *Q*-scores ≥30. Individual library sequence quality metrics were assessed by referencing the sequencing quality reports generated by analysis through the Qiagen CLC workbench program. For SARS-CoV-2 S gene NGS runs, up to 24 libraries can be sequenced on a single flow cell while the number of reads displayed in the trim summary section of the trim report should be ≥50,000 for each amplicon (before trimming of reads). Ninety-five per cent of nucleotide positions between 21615 and 25436 should have a coverage of 100×. SARS-CoV-2 whole virus was used as a positive control. The LOD with all replicates for all four (S1–S4) amplicons was 6,667 copies per ml.

##### Spike gene sequencing assay from Monogram Biosciences

Viral RNA from nasal swabs stored in Universal Transport Medium was extracted using the Kingfisher Flex platform. Following extraction, nested RT–PCR reactions were performed to amplify the entire SARS-COV-2 spike coding region. The resulting amplicon was purified and normalized before undergoing NGS library preparation (Kapa). Resulting barcoded libraries were normalized, pooled and underwent 2 × 150-bp paired-end sequencing on the Illumina MiSeq platform. FASTQ files were analyzed using a semiautomated bioinformatics pipeline. Briefly, reads were quality trimmed and overlapping paired reads joined. Reads were aligned in a codon-aware manner that maintains reading frame through the S gene. Codon and amino acid variants were determined and reported along with consensus sequences. Results were reviewed to ensure appropriate coverage levels, and quality metrics were obtained for each run and each individual sample. A sensitivity to amplification of ~1,000 copies per ml and a minor variant detection threshold of 3% were validated.

##### Whole-genome sequencing assay from Eurofins Viracor

Viral RNA from nasal swabs was extracted using the Kingfisher Flex platform and GSD NovaPrime RNA extraction kit. Extracted RNA was used as template in a one-step RT–PCR for cDNA synthesis. Each cDNA was subjected to amplification using ARTIC SARS-CoV-2 Primer Pools. These primer pools are designed to amplify approximately 90 amplicons each, with each amplicon averaging ~400 bp. Mapping these amplicons to a reference sequence illustrates the ‘tiled’ approach used for primer design, resulting in coverage of the entire SARS-CoV-2 genome. Purification of ARTIC PCR reactions was performed manually with Beckman-Coulter SPRIselect magnetic beads. The concentration of amplified amplicons in each sample was quantified using the Qubit FLEX fluorometer. Preparation of libraries was performed using the NEBNext Ultra II FS library prep kit in conjunction with the BRAVO liquid handling platform. Automated purification of library reactions was performed using the Agilent BRAVO liquid handler and Beckman-Coulter SPRIselect magnetic beads. Fragment size distribution of the final pooled library was confirmed using either the Agilent TapeStation 4200 or ThermoFisher BioAnalyzer 2100 DNA fragment analyzer before preparation for sequencing. Pooled libraries were denatured and sequenced on either the NextSEQ 500 or 550 instrument using a NextSEQ Mid Output 500/550 flow cell and reagents running a 2 × 150-cycle paired-end sequencing protocol. A Twist SARS-CoV-2 RNA positive control was processed in parallel with each verification run for positive control of RT–PCR, ARTIC PCR amplification and library preparation. The LOD for SARS-CoV-2 whole-genome sequencing was determined to be 100 copies per ARTIC PCR assay reaction.

#### Variant data analyses

SARS-CoV-2 variants in the study population were assessed based on amino acid mutations in the spike protein relative to the reference strain (spike mutations). For each of the three sequencing datasets (spike gene sequencing from Eurofins Viracor, spike gene sequencing from Monogram Biosciences (LabCorp) and whole-genome sequencing from Eurofins Viracor), spike protein amino acid mutations were acquired directly from the sequencing service provider. For each specimen, a single spike haplotype was designated as the ordered set of spike mutations. The same analysis was performed for the global SARS-CoV-2 genomic database available from GISAID^30,36^. Pango Lineages^35^ were inferred for clinical specimens in two ways. First, they were inferred from the lineage annotations of matching spike haplotypes included in the GISAID database. In cases when a single spike haplotype was associated with more than one Pango Lineage in GISAID, the dominant lineage was used. Second, Pango Lineages were inferred based on the presence of core backbone mutations from CDC variants in a specimen’s spike haplotype^28^. To assess the relative prevalence of select lineages, the full clinical and GISAID datasets were subset to include only records annotated with the selected lineages. Prevalence for each selected lineage was then computed as the percentage of total records for a given month within each data subset.

##### Variant-specific vaccine efficacy of mRNA-1273: statistical analysis method

For specific viral variants with a sufficient number of variant cases during the blinded phase of the study, the competing risk method was used to estimate the VE of mRNA-1273. COVID-19 cases of specific variant were considered as cases, and COVID-19 cases of variants other than that of interest were considered as competing events in this analysis. VE was defined as 1 – hazard ratio (mRNA-1273 versus placebo), and 95% CIs were estimated using Fine and Gray’s subdistribution hazard model with disease cases as competing events and the treatment group as a covariate, adjusting for stratification factor. Person-years was defined as total years from randomization date to the earliest among date of symptomatic SARS-CoV-2 infection, date of asymptomatic SARS-CoV-2 infection, last date of study participation and efficacy data cutoff date. Incidence rate was defined as the number of participants with an event divided by the number of participants at risk and adjusted by person-years (total time at risk) in each treatment group. The 95% CI was calculated using the exact method (Poisson distribution) and adjusted by person-years.

##### SARS-CoV-2 RT–PCR test (Eurofins Viracor)

SARS-CoV-2-specific RT–PCR assay was used to detect SARS-CoV-2 RNA in upper respiratory (nasal/nasopharyngeal wash and swab) and bronchoalveolar lavage (BAL) samples. This assay, performed by Eurofins Viracor, provides qualitative detection of RNA from SARS-CoV-2 virus in specimens collected from individuals meeting SARS-CoV-2 virus clinical criteria (https://www.fda.gov/media/136740/download). Briefly, the SARS-CoV-2 RT–qPCR assay was performed as a multiplex reaction with the MS2 internal control assay. Oligonucleotide primers and TaqMan probes were used for the detection of two regions of the viral NP protein gene region of SARS-CoV-2, and an internal extraction and amplification control target (the RNA bacteriophage MS2) was used. The LOD for this assay was determined to be 73 copies per ml for BAL, nasal wash and nasopharyngeal swab, with Ct = 38 being the cutoff for a positive result. SARS-CoV-2 RT–qPCR was also used for quantitative detection of viral RNA in both nasopharyngeal swabs and saliva samples. The LOD is 299 copies per ml and lower level of quantitation is 714 copies per ml.

#### Detection of respiratory pathogens

Participants with symptoms of a respiratory illness were advised to contact the clinical site within 72 h of onset. Such visits defined the day 1 illness date and triggered SARS-CoV-2 molecular testing via a nasopharyngeal swab that included testing for additional respiratory pathogens (BioFire RP2) and a 28-day follow-up period with periodic sampling (SARS-CoV-2 RT–PCR) also at days 3, 5, 7, 9, 14, 21 and 28 using saliva samples.

##### BioFire RP2 (Eurofins Viracor)

Respiratory pathogens were detected using BioFire RP2, a diagnostic multiplexed nucleic acid test intended for the simultaneous qualitative detection and differentiation of nucleic acids from 20 viral and bacterial respiratory organisms. The disposable closed system pouch was run on the Filmarray Torch system that lyses samples, extracts and purifies all nucleic acids and performs nested multiplex PCR. Endpoint melting curve data were used to detect target-specific amplicons and analyze data to generate a result for each analyte. Nasopharyngeal swabs were used as qualitative diagnostic assays for the detection of 20 different viruses and bacteria associated with respiratory tract infection. Assay results are provided as negative or positive for each pathogen in the BioFire Respiratory.

### Reporting Summary

Further information on research design is available in the Nature Research Reporting Summary linked to this article.

## Online content

Any methods, additional references, Nature Research reporting summaries, source data, extended data, supplementary information, acknowledgements, peer review information; details of author contributions and competing interests; and statements of data and code availability are available at 10.1038/s41591-022-01679-5.

## Supplementary information

Supplementary Information Supplementary Tables 1–9, Fig. 1 and COVE trial protocol.

Reporting Summary

## Data Availability

The variant sequence prevalence data are available in the GISAID repository (https://covariants.org/per-country). Because the trial is ongoing, access to patient-level data presented in this article (viral copy, variant VE and respiratory pathogens) and supporting clinical documents with external researchers who provide methodologically sound scientific proposals will be available upon request and subject to review once the COVE trial is complete. Such requests can be made to Moderna Inc., 200 Technology Square, Cambridge, MA 02139, USA. A materials transfer and/or data access agreement with the sponsor will be required for accessing of shared data. All other relevant data are presented in the paper. The protocol is available in the Supplementary Information: Clintrials.gov. NCT04470427.
