# Supplementary material for: Initial analysis of viral dynamics and circulating viral variants during the mRNA-1273 Phase 3 COVE trial
Source: Nat Med. 2022 Feb 10;28(4):823–30. doi: 10.1038/s41591-022-01679-5 (PMC9018421; doi:10.1038/s41591-022-01679-5)
Supplement: Supplementary file 2 — Reporting Summary [file 41591_2022_1679_MOESM2_ESM.pdf]

nature research

Corresponding author(s): Rolando Pajon

Last updated by author(s): December 15, 2021

Reporting Summary

Nature Research wishes to improve the reproducibility of the work that we publish. This form provides structure for consistency and transparency in reporting. For further information on Nature Research policies, see our Editorial Policies and the Editorial Policy Checklist.

Statistics

For all statistical analyses, confirm that the following items are present in the figure legend, table legend, main text, or Methods section.

n/a	Confirmed	
☐	☒	The exact sample size (*n*) for each experimental group/condition, given as a discrete number and unit of measurement
☒	☐	A statement on whether measurements were taken from distinct samples or whether the same sample was measured repeatedly
☐	☒	The statistical test(s) used AND whether they are one- or two-sided<br>*Only common tests should be described solely by name; describe more complex techniques in the Methods section.*
☐	☒	A description of all covariates tested
☐	☒	A description of any assumptions or corrections, such as tests of normality and adjustment for multiple comparisons
☐	☒	A full description of the statistical parameters including central tendency (e.g. means) or other basic estimates (e.g. regression coefficient) AND variation (e.g. standard deviation) or associated estimates of uncertainty (e.g. confidence intervals)
☐	☒	For null hypothesis testing, the test statistic (e.g. *F*, *t*, *r*) with confidence intervals, effect sizes, degrees of freedom and *P* value noted<br>*Give P values as exact values whenever suitable.*
☒	☐	For Bayesian analysis, information on the choice of priors and Markov chain Monte Carlo settings
☒	☐	For hierarchical and complex designs, identification of the appropriate level for tests and full reporting of outcomes
☒	☐	Estimates of effect sizes (e.g. Cohen's *d*, Pearson's *r*), indicating how they were calculated

Our web collection on statistics for biologists contains articles on many of the points above.

Software and code

Policy information about availability of computer code

Data collection	TapeStation Analysis Software 4200 Controller A.02.02 and Qiagen CLC Genomics Workbench software v20.0.1 were used for the analysis of spike-gene sequencing.
Data analysis	Statistical Analysis System (SAS) Version 9.4 was used (SAS Institute Inc., Cary, North Carolina).

For manuscripts utilizing custom algorithms or software that are central to the research but not yet described in published literature, software must be made available to editors and reviewers. We strongly encourage code deposition in a community repository (e.g. GitHub). See the Nature Research guidelines for submitting code & software for further information.

Data

Policy information about availability of data

All manuscripts must include a data availability statement. This statement should provide the following information, where applicable:

- Accession codes, unique identifiers, or web links for publicly available datasets
- A list of figures that have associated raw data
- A description of any restrictions on data availability

The GISAID dataset analyzed during the current study is available at https://covariants.org/per-country. As the trial is ongoing, access to patient-level data and supporting clinical documents with qualified external researchers may be available upon request and subject to review once the trial is complete.

Field-specific reporting

Please select the one below that is the best fit for your research. If you are not sure, read the appropriate sections before making your selection.

☒ Life sciences ☐ Behavioural & social sciences ☐ Ecological, evolutionary & environmental sciences

For a reference copy of the document with all sections, see nature.com/documents/nr-reporting-summary-flat.pdf

Life sciences study design

All studies must disclose on these points even when the disclosure is negative.

Sample size

For the analysis of viral copy, of 799 adjudicated cases in the trial, 701 (48 in the mRNA-1273 and 653 in the placebo groups) with no evidence of infection through day 57 and were included in the analysis. A MMRM analysis was performed comparing absolute and change from baseline in log10 viral load between vaccinated and placebo participants from nasopharyngeal swabs at day 1 of illness and in saliva samples through days 3, 5, 7, 9, 14, 21, and 28 of illness. Only participants with a quantitative result available at the day 1 illness nasopharyngeal swab were included in the MMRM analysis, and included a total of 36 participants in the mRNA-1273 and 595 in the placebo groups. For SARS-CoV-2 genome sequencing, there were a total of 1006 samples available (142 for mRNA-1273 and 864 for placebo). Sequences were obtained 832 different samples (754 placebo and 78 mRNA-1273), corresponding to 791 trial participants (720 from placebo and 71 from mRNA-1273). Vaccine efficacy of variants was based on 799 adjudicated cases in the trial (744 for placebo and 55 for mRNA-1273).

Data exclusions

In the analysis of viral load, of the 799 adjudicated cases, only those participants who had no evidence of infection through day 57 were included.

Replication

For clinical trial data, data was collected once for each timepoint. Replicate samples were run for Intra- and inter-assay precision in the Eurofins Viracor assay as described (https://www.fda.gov/media/136740/download).

Randomization

As described previously in the COVE trial, Participants were randomly assigned in a 1:1 ratio to receive two doses of the mRNA-1273 vaccine (100 µg) or placebo and were stratified according to age and Covid-19 complications risk criteria (≥18 to <65 years and not at risk, ≥18 to <65 years and at risk, and ≥65 years).

Blinding

Vaccine preparation and administration were performed by study team pharmacists who had no other role in the conduct of the study. Following injection, all study team members and participants were blinded to treatment. Access to the allocation code was strictly controlled at the pharmacy. Designated team members within the sponsor, Moderna, were unblinded to the data for interfacing with the regulatory agencies and the data and safety monitoring board (DSMB) but had no part in assessment prior to database lock; all other trial staff and participants remained blinded to treatment assignments until the blinded part of the trial concluded. The DSMB reviewed group level efficacy data and unblinded participant safety data.

Reporting for specific materials, systems and methods

We require information from authors about some types of materials, experimental systems and methods used in many studies. Here, indicate whether each material, system or method listed is relevant to your study. If you are not sure if a list item applies to your research, read the appropriate section before selecting a response.

Materials & experimental systems

n/a	Involved in the study	
☒	☐	Antibodies
☒	☐	Eukaryotic cell lines
☒	☐	Palaeontology and archaeology
☒	☐	Animals and other organisms
☐	☒	Human research participants
☐	☒	Clinical data
☒	☐	Dual use research of concern

Methods

n/a	Involved in the study	
☒	☐	ChIP-seq
☒	☐	Flow cytometry
☒	☐	MRI-based neuroimaging

Human research participants

Policy information about studies involving human research participants

Population characteristics

The mean age was 49 years (range 18-87 years) and 50% were female. In the mRNA-1273 group, more participants had chronic lung disease, significant cardiac disease, severe obesity and liver disease, lower incidences of diabetes and HIV, and a higher mean of body mass index than the placebo group.

Recruitment

In the COVE trial, from July 27 to October 23, 2020, participants were enrolled and randomized. Participants were recruited to the sites through largely three ways, (1) review of unit past participant logs for eligible individuals based on the inclusion

and exclusion criteria, (2) public advertisements using social media, and (3) referrals from primary care physicians. As such, there are not believed to be any self-selection biases that would impact results.

Ethics oversight	The COVE trial is conducted in accordance with the International Council for Technical Requirements for Registration of Pharmaceuticals for Human Use, Good Clinical Practice Guidance, and applicable government regulations. The central Institutional Review Board/Ethics Committee, Advarra, Inc., 6100 Merriweather Drive, Columbia, MD 21044 approved the protocol and consent forms. All participants provided written informed consent.

Note that full information on the approval of the study protocol must also be provided in the manuscript.

Clinical data

Policy information about clinical studies

All manuscripts should comply with the ICMJE guidelines for publication of clinical research and a completed CONSORT checklist must be included with all submissions.

Clinical trial registration	clintrials.gov NCT04470427
Study protocol	Protocol was provided at submission as supplemental information and is posted online with publication of the article.
Data collection	The COVE study was conducted at 99 sites across the US. These sites and team are listed in the supplement. In the trial, from July 27 to October 23, 2020, participants were enrolled and randomized, and the study is ongoing until 2 years of follow-up are completed.
Outcomes	In this analysis of the COVE trial, pre-specified outcomes assessed included evaluation of viral load and duration of detectable viral RNA in adjudicated Covid-19 cases, genomic sequencing of viral variants in all Covid-19 and adjudicated Covid-19 cases, burden of disease and infection in adjudicated cases, and vaccine efficacy against variants assessed in adjudicated Covid-19 cases in the per-protocol set from the blinded portion of the study (data cut-off March 26th, 2021). Following amendment (December 23rd, 2020) of the COVE phase 3 study, the open-label phase of the trial was initiated and participants in the placebo arm started to receive the mRNA-1273 vaccine. Sequence data for viral variants and also assessment of the presence of other respiratory pathogens extended into the time-frame of the open-label part of the study.
